# How Dairy Cows Are Culled from Freestall-Housed Dairy Herds in Wisconsin

**DOI:** 10.3390/ani16020238

**Published:** 2026-01-13

**Authors:** Kaitlin I. Buterbaugh, Thomas B. Naze, Nigel B. Cook

**Affiliations:** School of Veterinary Medicine, University of Wisconsin-Madison, 2015 Linden Drive, Madison, WI 53706, USA; kbuterbaugh@wisc.edu (K.I.B.); tbnaze1@gmail.com (T.B.N.)

**Keywords:** culling, turnover, decision-making, herd management

## Abstract

Efforts to improve efficiency and profitability on dairy farms have renewed focus on how culling practices affect herd sustainability and economic outcomes. This study surveyed decision-makers on 60 high-producing, freestall-housed dairy farms in Wisconsin, with a mean (SD) turnover rate of 36.0 (8.0)%. Using a structured questionnaire, we examined herd management, culling criteria, and motivations. Most farms (93%) used on-farm management systems to guide culling, yet only 48% used designated reports, relying instead on individual cow records. Milk production, infertility, and somatic cell count were the top culling criteria, with high milk yield cited as the most difficult factor in removal decisions. While 54% recorded the most obvious reason for culling, only 7% documented multiple causes. Cull cows were typically transported by third parties; 80% farms sent cows directly to slaughter, while 52% sent them to auction. One-third of farms sold cows for continued dairy use. Euthanasia was performed on 93% of farms, mostly by employees, with minimal veterinary input. The study aimed to investigate producer perspectives on the culling decision-making process on commercial dairy farms. The findings highlight opportunities for improved veterinary involvement and the use of structured herd-level reports to support more strategic culling decisions.

## Simple Summary

Dairy farmers regularly remove cows from their herds, a process known as culling, to maintain productivity and manage herd health. However, little is known about how these decisions are actually made on farms. This study surveyed 60 dairy farms in Wisconsin to understand how farmers decide which cows to remove. Most farms used computer records to help make decisions, but typically relied on individual cow data rather than organized reports. Farmers often prioritized milk production, fertility, and udder health when choosing cows to cull, and many found it difficult to remove cows that still produced a lot of milk. While most farms sent cows directly to slaughter, some also sold them to other farms or sent them to auction. Decisions about euthanasia were usually made by farmers without input from veterinarians. The study found that record-keeping was inconsistent, and few farms recorded more than one reason for removing a cow. These findings suggest that better tools and more veterinary involvement could help farmers make more informed and humane decisions. Improving how culling decisions are made could benefit animal welfare, farm efficiency, and the environment.

## 1. Introduction

As dairy farms strive for efficiency, profitability, and sustainability, the implications of culling practices have garnered increased attention recently, given the current reduction in the national inventory of heifer replacements in the US [1]. At the same time, there is an ongoing discussion regarding the mean productive life and animal welfare concerns surrounding the relatively short lifespan of dairy cows in herds in progressive dairy industries around the world (typically 4–6 years), relative to an expected lifespan of up to 20 years [2]. The increased greenhouse gas emissions from the rearing of excessive numbers of heifers are also a concern, impacting dairy sustainability [3].

Culling, defined as the removal of cows from the herd due to sale, slaughter, salvage, or death, is a routine practice [4]. When performed appropriately, culling is the process of improving the herd by replacement [5], through the removal of a poor performing animal and its substitution with a fit, better performing younger animal.

In the US, DHIA organizations measure the amount of culling using the herd annual turnover rate—defined as the number of cows leaving the herd over a 12-month period, divided by the average number of cows in the herd (milking and dry) over the same time period [4]. In the US, herd turnover rate has typically averaged around 35% [6,7]—implying that mean productive life is 100/35 = 2.9 lactations, or 4.9 years, assuming a mean age at first calving of around 24 months.

Mean turnover rate is a function of replacement supply, given that US dairy farmers have traditionally bred the entire herd to a Holstein bull using conventional unsexed semen, creating ~36 heifers for every 100 cows after losses during the rearing process are accounted for (assuming 93% of cows become pregnant, a 48% female sex ratio, and a 20% loss from birth to calving = (100*0.93)*0.48 = 45 heifers born 45*0.80 = 36 heifers entering a herd of 100 cows). Interestingly, breeding management in dairy herds has changed over the last decade. Simultaneously, we have observed the adoption of sexed Holstein semen for breeding of replacement heifers from younger cows and heifers in the herd, and the use of conventional beef semen to deliver a beef cross calf of higher value, typically from older cows in the herd [8]. The adoption of this technology may have resulted in a reduction in available replacement heifers in some herds, contributing to the decline in the national inventory of heifers and increased prices.

Some have argued that high turnover rates and shorter productive lives are unsustainable, being reflective of poor health and welfare, causing a negative environmental impact of rearing high replacement numbers, and economic loss of failing to recoup heifer rearing costs [2]. However, the reality is that herd turnover rate serves as a poor indicator of herd performance and animal welfare, since it varies widely among herds with similar production levels. Turnover rate may be lower in herds with excellent health and performance, but also in herds with significant health challenges, and higher in otherwise healthy herds with a robust supply of replacements and good fertility programs [9]. Indeed, in Sweden, Owusu-Sekyere et al. noted that relatively short longevity was not a result of health and welfare problems, but a function of farmers’ investment decisions and herd-specific management practices [10], while in the Netherlands, Han et al. concluded that herd longevity may chiefly be determined by farmers’ attitudes and strategic management [11].

The most common reasons for culling in US dairy herds are reported to be injury, low milk production, reproductive problems, mastitis, and death [6,7]. These problems may occur as a result of genetics, farm management practices, environmental risk factors, infectious agents, or overall herd health management. Herd-level factors such as overstocking, the supply of eligible replacements, and herd labor capacity can also influence a cow’s risk of removal from the farm, as can macroeconomic factors such as milk price, feed cost, cull price, and heifer cost.

Moreover, record-keeping of culling practices is relatively poor and subject to a great margin of human error. USDA reports suggest that 78% of dairy operations use a record-keeping system to track cull cow sales [12]. Even on operations that track sales of culled animals, the quality of cull cow data is inconsistent and does not tell the story of how these decisions are being made. Less than 25% of dairy herds rely on input from their veterinarian to make culling decisions [12]. The culling process, therefore, is almost entirely dictated by farm owners and managers. While prior research has been conducted to examine the reasons that cows are culled from dairy farms, there is little qualitative research that details how farmers are making culling decisions and what factors are prioritized when determining which cow to cull from the herd.

The aim of this study was, therefore, to investigate the processes involved in the selection of cows to be culled from the herd, in order to gather data on specific practices that may be impacted in US dairy herds. To our knowledge, this is the first such attempt to investigate these practices. This research addresses a critical gap in existing literature by focusing on the decision-making process itself, rather than merely the outcomes. We seek to provide insight into how farmers prioritize factors such as production, health, and labor when choosing to cull cows from the herd, as well as outlining the process they undergo to make culling decisions.

## 2. Materials and Methods

This study was performed on commercial dairy farms in Wisconsin and underwent review by the Institutional Review Board at the University of Wisconsin-Madison and was deemed ethical research.

Commercial dairy farms were identified using the Department of Agriculture, Trade and Consumer Protection (DATCP) Milk Producer License Holders list (consisting of 5717 farms), and a list of larger freestall-housed dairy herds that had participated in prior projects by the research team (253 farms). Farms were sorted by region of the state to facilitate visiting herds in clusters within each region (South-Central, South-East, South-West, East-Central, North-East, North-West, North, or Central), and each region consisted of between 3 and 50 farms. Farms were contacted via telephone and screened for on-farm record system use (records of cull cow reasons for removal were required), housing and management system (freestall housing), and willingness to participate in the study with an in-person visit to the farm.

Farms agreeing to participate were visited between May 2023 and April 2024 to collect data on the culling decision-making process by interviewing the senior decision-maker. Survey questions were developed to address the factors influencing culling decisions and the process that dairy producers undergo in deciding which cow to cull. The final survey consisted of 41 questions, formatted as multiple-choice or open-ended responses (Supplement S1). Questions were divided into the following categories: general management and facilities, culling process for sold cows, removal process for dead cows, the breeding program, and general questions regarding culling approach and philosophy.

The survey was conducted at the farm by 2 members of the study team using a custom Access (Microsoft Corp., Redmond, WA, USA) database and a physical copy of the survey. Responses were verified between the 2 surveyors upon completion of the survey to ensure quality and accuracy of responses, and the data were entered into Excel (Microsoft Corp., Redmond, WA, USA) for analysis.

A consultant backup of the on-farm record system was retrieved from the herd management software. Individual herd disposal codes were recorded at the time of backup retrieval for herds utilizing DairyComp305 (DC305 version 24; Valley Ag Software, Tulare, CA, USA), as individual codes were not transferable in the consultant backup.

Frequency analyses were conducted on the survey responses to yield descriptive statistics using pivot tables and R Studio version 3.6.3 (Posit PBC, Boston, MA, USA). Open-ended responses were categorized by common themes.

## 3. Results and Discussion

In total, 60 farms were surveyed, with a mean (SD) herd size of 1333 (1125) of predominantly Holstein breed cows and a mean (SD) milk production of 42.3 (4.1) kg/d, with 88.3% of herds milking cows at least 3 times a day and 11.7% milking twice daily. Other herd performance data are provided in Table 1, with mean, median, and range. Forty-six herds (76.7%) utilized DC305 management software, six (10.0%) utilized PCDart (DRMS, Raleigh, NC, USA), three (5.0%) utilized BoviSync (BoviSync, Fond Du Lac, WI, USA), and five herds used a proprietary AMS software or other type of system (Table 2).

Our survey determined the mean (SD) turnover rate to be 36.0 (8.0)% across all herds, and 33.0 (6.0)% when dairy sales were removed. In a review published in 2020, De Vries and Marcondes found the average turnover rate to be 34% without dairy sales [7], while comparatively, the average turnover rate for the period of 1993–1999, excluding cattle sold for dairy, was 31.6% [6]. It would therefore appear that in the US, over an almost 30-year period, there was little change in the overall herd turnover rate. Mean (SD) death rate was 5.0 (2.0)%, which compares with a published mortality rate of 5.7% in US dairy herds [9], suggesting little to no change in death rate in the last decade.

In the current study, two-thirds of herds (66.7%) culled cows as needed, whether due to space restrictions, the condition of the animal, or other factors forcing removal, with little to no prior planning. However, one third of herds (33.3%) culled cows at a fixed interval, most commonly selecting cows for removal on a weekly basis. Interestingly, one-third of farms in this study (33.3%) sold cows to another farm as a dairy sale to continue their productive life. This agrees with a Canadian study that found that 38% of farms reported dairy sales [13].

**Figure 1:**
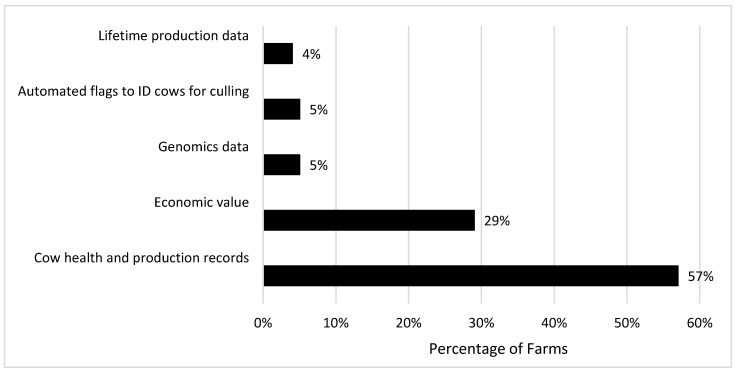
Farm response to the question “What would help you choose a cow to be culled?”.

When asked how they decide which cow to cull from the herd, the majority of herds (93.3%) stated that they utilize records from an on-farm management system. A total of 57% of herds responded with ‘good records’ in response to the question “What would help you choose a cow to be culled?” (Figure 1), but surprisingly, given this response, only 48.3% of farms utilized a designed report obtained from the on-farm management system to help make their decision, while the remaining herds relied solely on individual cow records. Farms that did utilize herd-level reports most commonly utilized parameters including milk production, somatic cell count, number of health events (predominantly lameness and clinical mastitis events), and reproductive status. Despite the most commonly used herd management system in the study (DC305) having an estimate of net present value of each cow in the herd (Cowval), and 29% of farms stating that they wanted an estimate of economic value to help choose a cow to cull, no farm responded that they utilized Cowval in their decision making, suggesting that this function is underutilized in the industry.

**Figure 2:**
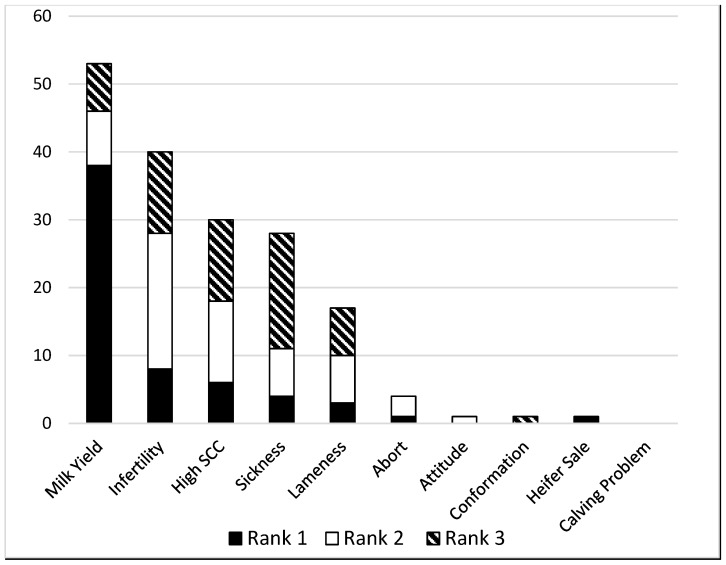
Count of stated rank priority for culling reasons given by 60 dairy farmers.

When asked to rank culling reasons by priority (1–3), milk production, infertility, somatic cell count, sickness, and lameness emerged as the most common choices (Figure 2). This is in agreement with Kulkarni et al., who also found reproduction, milk production, and udder health were prioritized as reasons for culling, but also included claw health, breeding value, and body conformation [14]. An Estonian study found the most common culling reasons to be feet/claw disorders, udder disorders, and metabolic and digestive disorders, rather than production and fertility, perhaps indicative of different challenges facing that industry [15].

**Figure 3:**
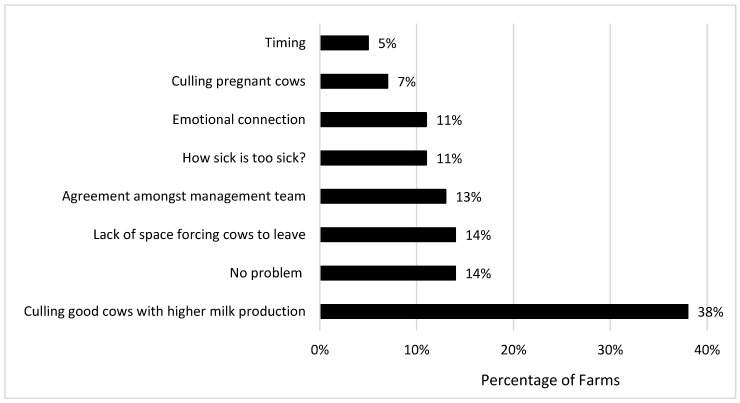
Farm response to the question “What do you find most challenging about deciding when to cull a cow?”.

When farmers were asked “What do you find most challenging about deciding when to cull a cow?” (Figure 3), 38% responded that culling good cows with higher milk production was the most challenging aspect of the decision, with other responses including lack of space, agreement with other managers, determining the severity of the illness and emotional connection with the animal. Only 14% of herds claimed that they did not have a problem selecting the cow.

**Figure 4:**
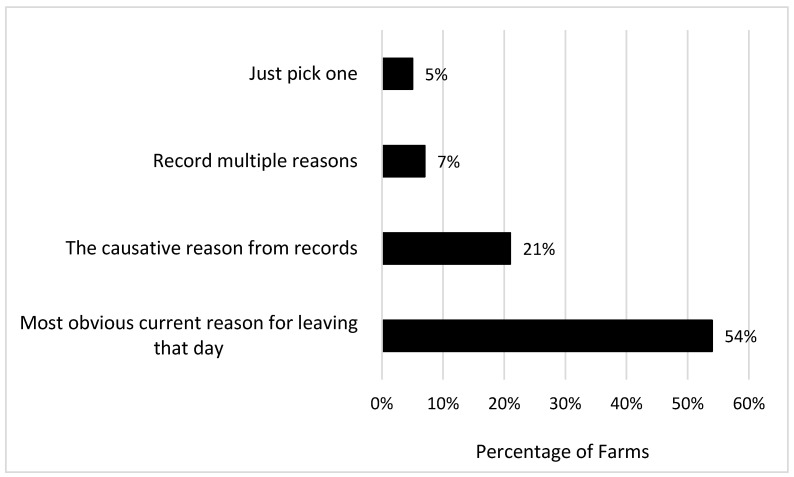
Farm response to the question “When there are multiple reasons, how do you pick a reason to record why the cow is culled?”.

When assigning a reason for removal, 54% of farms stated that they pick the most obvious current reason for leaving that day (Figure 4). However, 21% of farms try to choose the causative reason why the cow was leaving. This would potentially impact whether or not a cow was culled for low milk production, or the supposed reason for the low milk production. A small number of herds (7%) recorded multiple reasons, which has been a long-standing recommendation in the industry. Fetrow et al. suggested a two-tiered coding system for cow removals, where cow removals would be coded for the destination first (dairy sale, slaughter, or death), followed by the reason for removal, but this approach has not been adopted widely [4].

**Figure 5:**
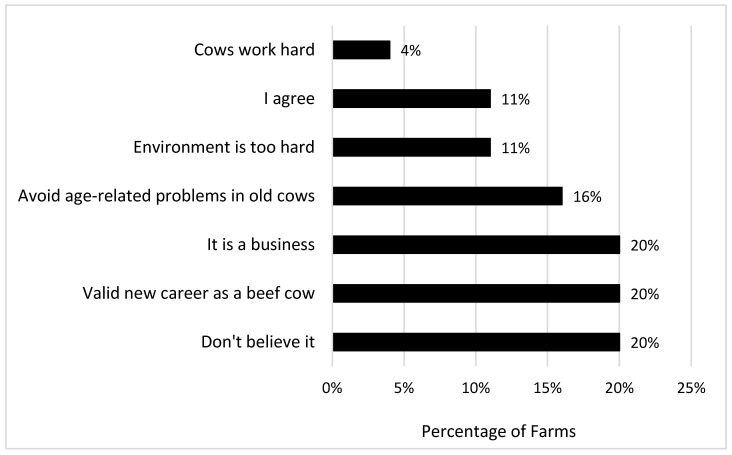
Farm response to the complaint that “cows live too short of a lifespan (less than 3 lactations)”.

Finally, when challenged with how to respond to the statement that “cows live too short of a lifespan (less than 3 lactations)”, a range of responses were obtained, with 20% of farmers responding that they ‘didn’t believe it’, or that ‘it was a business’ and that ‘culled cows had a new career as a beef cow’ (Figure 5). A smaller percentage agreed (11%) and also stated that the environment might be too hard for the cows (11%), and that they culled at a higher rate to avoid age-related problems (16%).

Cull cows were picked up by a third-party transporter on three-quarters of farms, whatever their final destination. Eighty percent of farms sent cattle directly to slaughter, while 51.7% of farms also sent cows to auction.

In making the decision to euthanize a cow, only 6.7% of farms relied on advice from their herd veterinarian, while 86.6% based their decision solely on the duration and severity of clinical signs in the animal. Euthanasia was performed by the majority of farms (93.3%), and it was most commonly performed by a farm employee (76.7%). Only 23.3% of herds stated that they had a dedicated fenced-in area for dead stock, indicating the potential for growth in the adoption of this practice. Cows that died or were euthanized on farm were most often picked up by a third party (76.7% of farms) rather than composted (21.7%) or incinerated on farm (1.7%), confirming the continued reliance on an independent rendering industry.

## 4. Conclusions

Based on the responses in this study, dairy producers describe culling cows primarily on an individual cow basis, ‘one cow at a time’, utilizing individual cow records rather than organized reports with data summaries, to select cows to cull when driven by space restrictions, the condition of the animal, or other factors forcing removal. This creates an opportunity going forward to develop a more organized, structured process for identifying cows for removal, incorporating milk production, reproduction, health, and economic outcomes in a planned manner. Few farms use the recommended approach of recording multiple reasons for cow removal. While the majority record the most obvious reason for leaving on that day, some attempt to select a causative reason, which impacts the quality of the records collected, explaining reasons for culling. The dairy industry relies heavily on third-party transportation of live and dead animals from the farm, and there is an opportunity for increased veterinary involvement in euthanasia decisions.

## Figures and Tables

**Table 1:** Summary annualized statistics of herd level variables on farms surveyed.

Variable	Mean	Median	SD	Minimum	Maximum
Average no. lactating cows in the herd	1333	986	1042	101	5414
Percent of cows in first lactation	34%	34%	5%	20%	45%
Average no. lactations per cow	2.93	2.86	0.77	1.75	6.67
Average days in milk (DIM)	191	189	21	163	286
Average age at first calving (months)	24	23	3.69	21	49
Average duration of dry period (days)	58	57	4.93	49	74
Average milk per cow per day (kg)	42	43	4.3	25	49
Average somatic cell count (‘000/mL)	161	140	84	57	506
Milk fat percent	4.23	4.23	0.33	3.72	5.38
Milk protein percent	3.27	3.25	0.14	3.04	3.82
Annual pregnancy rate (%)	31%	31%	6%	12%	43%
Turnover rate (%)	36%	35%	8%	15%	57%
Death rate (%)	5%	5%	2%	2%	11%
Turnover rate (less dairy sales) (%)	33%	33%	6%	23%	57%
Turnover rate < 60 DIM (%)	9%	8%	5%	2%	32%

**Table 2:** Descriptive summary of categorical responses by 60 dairy farmers to a questionnaire exploring current culling practices.

Variable	N (Farms/ Responses)	Levels/Categories	Counts (n)	Percent (%)
Marketing frequency	60	As needed	40	66.7
		At a fixed interval	20	33.3
Cows sent to auction	60	Yes	31	51.7
		No	29	48.3
Cows sent directly to slaughter	60	Yes	48	80.0
		No	12	20.0
Cows sold for dairy	60	Yes	20	33.3
		No	40	66.7
Transport of cull cows	60	Third party	38	63.3
		Originating farm	13	21.6
		Third party and originating farm	6	10.0
		Originating farm and purchasing farm	1	1.7
		Third party and purchasing farm	1	1.7
		Third party, originating farm, and purchasing farm	1	1.7
Culling information source	60	Dairy records system	56	93.3
		Farm workers	2	3.3
		Other	2	3.3
Use of a culling report	60	Yes	29	48.3
		No	31	51.7
Dead cow disposal	60	Pick up by a third party	46	76.7
		Compost	13	21.7
		Incinerate	1	1.7
Dead cow storage location	60	Just a place out of sight	30	50.0
		Dedicated location with fencing	14	23.3
		Compost pile	11	18.3
		No dedicated storage	5	8.3
Euthanasia performed on farm	60	Yes	56	93.3
		No	4	6.7
Individual responsible for euthanasia	60	Farm employee/owner	46	76.7
		Third party	6	10.0
		DVM	4	6.7
		Do not perform euthanasia on farm	4	6.7
Euthanasia decision	60	Based on duration and severity of clinical signs	52	86.7
		Advice from DVM	4	6.7
		Do not perform euthanasia on farm	4	6.7
Stated culling report parameters used	77	Milk	34	44.2
		Somatic cell count	15	19.5
		Health	7	9.1
		Repro	7	9.1
		Do Not Breed	4	5.1
		Days in Milk	3	3.9

## Data Availability

The raw data supporting the conclusions of this article will be made available by the authors upon request.

## Supplementary Materials

The following supporting information can be downloaded at: https://www.mdpi.com/article/10.3390/ani16020238/s1, Supplement S1: Farm Questionnaire.

## Abbreviations

The following abbreviations are used in this manuscript:

SD	Standard Deviation
USDA	United States Department of Agriculture
DHIA	Dairy Herd Improvement Association
DC305	DairyComp305 (herd management software)
AMS	Automated Milking System
DVM	Doctor of Veterinary Medicine
