# Supplementary material for: How Dairy Cows Are Culled from Freestall-Housed Dairy Herds in Wisconsin
Source: Animals (Basel). 2026 Jan 13;16(2):238. doi: 10.3390/ani16020238 (PMC12837832; doi:10.3390/ani16020238)
Supplement: Supplementary file 1 [file animals-16-00238-s001.zip › animals-4002239-supplementary.pdf]

**Date:**
**Farm Name:**
**Farm Representative (First and Last Name):**
**Farm Address (Street, City, State and Zip):**
**Phone1:** **Phone2:**
**Survey Administrator:**
**Farm Size: (milking)** **(milking plus dry)**
**Who is responsible for Culling Decisions:**
**Who is responsible for Culling Reason Records:**
**Who is the veterinarian that you work with/what clinic are they with?**
General management and facilities
What is the predominant breed in your herd?
a. Holstein
b. Jersey
c. Cross breed
d. Other
Please describe other:____
Is the dairy F.A.R.M. certified? YES / No
a. F.A.R.M.
b. ProAction
c. MMAP
d. Other
Please describe other:____
What is the form of housing used by the majority of the milking cows?
a. Freestall
b. Bedded Pack
c. Dry lot
d. Other
Please describe other:____

What is the milking frequency used for the majority of the milking cows?
a. 2x
b. 3x
c. 4x
d. Other. Please describe____.
How are the majority of cows milked?
a. Parlor
b. Robot
What bedding surface do you use?
a. Mattress
b. Deep bedding
What type of bedding do you use?
a. Fresh sand
b. Recycled sand
c. Manure solids
d. Organics
What on farm records system is used to manage the herd?
a. DC305
b. PCDart
c. DHI
d. Bovisynch
e. Other
Please describe other:____
How are animals grouped (check all that apply)
a. DIM
b. Pregnancy status
c. Size
d. Age
e. Other. Describe ____
Are any heifers reared off farm?
a. Yes
b. No
If yes, what approx percentage? ____

Do any animals get outdoor (pasture or dry lot) access?
a. Yes
b. No
Which animals get outdoor access? Check all that apply.
a. Replacement heifers
b. Fresh animals
c. Mid to late lactation animals
d. Dry cows
Is the herd actively expanding in herd size over the last year?
a. Yes
b. No
General Management and Facility Notes:

Culling Process (SOLD cows)

How frequently do you market cows?
a. As needed
b. At a fixed interval
Please describe interval:____
Where do cows go when they leave the farm?
a. Auction
b. Slaughterplant
c. Another dairy farm
d. Other
Please describe:____
Who is responsible for transportation?
a. The purchasing farm
b. This farm
c. A third party
Please describe third party:____
What is the most common determinant of the timing for when a cow is to be culled from the farm?
a. Lack of space
b. Disease
c. Injury
d. Infertility/Repro
e. Poor milk yield
f. Other
Please describe other:____
What information source is used to assist in determining which cow to cull?
a. Farm workers
b. Dairy records system - DHI DC305
c. Herd consultants
d. Other

Please describe:____
Are dairy records reports used to assist in the culling decision?
a. Yes
b. No
Which dairy records report commands are used?
a. Enter commands or print off reports and collect
Which culling reason do you prioritize in order of importance when selecting a cow to be culled?
a. Milk yield
b. Infertility
c. High SCC/Chronic mastitis
d. Lameness
e. Attitude and safety
f. Sickness
g. Conformation
h. Abort
i. Heifer sale
j. Calving problem
What information do you record?
a. SOLD event
b. Disposal Code (DCAR)
c. Culling reason
d. Back tag number
e. Other
Please describe:____

Culling Process (Sold) Notes:

Culling Process (DIED cows)

Where do the dead cows go?
a. Compost on farm
b. Picked up by third party
Describe third party:
Cost per animal:
Is there a dedicated storage area designed for dead cows on farm before pick up?
a. Yes - dedicated location on farm with fencing
b. Yes, Just a place out of sight, but it's the same spot every time
b. No
Do you euthanase cows on farm?
a. Yes
b. No
Who euthanizes cows on the farm?
a. Farm employee/owner
b. DVM
c. Third party
How is euthanasia performed?
Who is responsible for euthanasia training on farm?
How do you decide when to euthanize a cow?
a. Advice from DVM
b. Based on duration and severity of clinical signs eg down cow
c. Feedback from caregivers
d. Concern regarding auditing
e. Trucker refuses to transport

Culling Prcess (Dead) Notes:

Breeding Program - Bull choice

For breeding age nulliparous breeding age (L0) heifers, what semen type is predominantly used:
a. Sexed/Holstein %
b. Sexed/Beef %
c. Conventional/Holstein %
d. Conventional/Beef %
e. Embryos %
For first lactation heifers (L1), what semen type is predominantly used:
a. Sexed/Holstein %
b. Sexed/Beef %
c. Conventional/Holstein %
d. Conventional/Beef %
e. Embryos %
For mature/multiparous cows (L2+), what semen type is predominantly used:
a. Sexed/Holstein %
b. Sexed/Beef %
c. Conventional/Holstein %
d. Conventional/Beef %
e. Embryos %
Do you use a breeding manager - determines which semen to use for each cow based on genomics?

Breeding Program Notes:

Culling (SOLD cows) - open ended questions

How do you define a cull cow?
What do you find most challenging about deciding which cow should be culled and when?
What would help facilitate the choice of cow to be culled?
How do you decide the reason for a cow being culled?
When there are multiple reasons for culling a cow, how do you choose the reason you record?

How do you respond to the complaint that cows live for less than 3 lactation cycles on average and that is too short?
Culling Questions Notes:
